# Environmental Contaminants and Congenital Heart Defects: A Re-Evaluation of the Evidence

**DOI:** 10.3390/ijerph15102096

**Published:** 2018-09-25

**Authors:** Rachel Nicoll

**Affiliations:** Department of Public Health and Clinical Medicine, Umeå University, SE 901-87 Umeå, Sweden; rachel.nicoll@umu.se; Tel.: +44-7917-787350

**Keywords:** congenital heart defects, environmental toxins, smoking, air pollution, disinfectant byproducts, waste sites, toxic metals, persistent organic pollutants, pesticides

## Abstract

Congenital heart defects (CHDs) are a common birth defect of largely unknown etiology, with high fetal and neonatal mortality. A review of CHDs and environmental contaminant exposure found that meta-analyses showed only modest associations for smoking, vehicle exhaust components, disinfectant by-products and proximity to incinerators, with stronger results from the newer, larger and better quality studies masked by the typical absence of effect in older studies. Recent studies of exposure to agricultural pesticides, solvents, metals and landfill sites also showed associations. Certain contaminants have been associated with certain CHDs, with septal defects being the most common. Frequent methodological problems include failure to account for potential confounders or maternal/paternal preconception exposure, differences in diagnosing, defining and classifying CHDs, grouping of defects to increase power, grouping of contaminants with dissimilar mechanisms, exclusion of pregnancies that result in death or later life diagnosis, and the assumption that maternal residence at birth is the same as at conception. Furthermore, most studies use measurement estimates of one exposure, ignoring the many additional contaminant exposures in daily life. All these problems can distort and underestimate the true associations. Impaired methylation is a common mechanism, suggesting that supplementary folate may be protective for any birth defect.

## 1. Introduction

Congenital heart defects (CHDs) are among the most common of the severe congenital abnormalities, accounting for around one third of all birth defects, and are a leading cause of spontaneous abortion, stillbirth and neonatal and infant mortality and the underlying reason for many medical abortions [1,2,3]. Approximately 30% of infants who die at birth are found to have a CHD [4] and in some studies they are present in approximately 1% of live births [5], although a UK investigation showed that in almost 700,000 live births, 6.4% had CHDs, 15% of which were life threatening; only 70% were diagnosed either prenatally or postnatally before discharge from hospital, leaving 30% to be diagnosed once medical problems became obvious or on death [6]. Both pre- and full-term infants with cardiovascular defects have a significantly increased mortality rate [3]. Children who survive may be left with physical, developmental and cognitive problems which can require lifelong medical treatment. While around 30% of all CHDs can be attributed to chromosomal or single gene disorders or other known causes, the cause of the remaining 70% remains unknown [7]. Many environmental contaminants have now been classified as endocrine disruptors [8] and may also act as teratogens through that mechanism, playing an important role in CHD causation. It has also now become widely recognised that mothers share their chemical load with the fetus and while breast feeding [9].

This review covers studies of human exposure to the normal levels of contaminants which might be found in the environment and their association, if any, with CHDs. Exposures studied include parental smoking, external air pollution, pesticides, contaminated drinking water, solvents, landfill sites, incinerators, toxic metals and other persistent organic pollutants. It also looks at potential mechanisms of effect and discusses the frequent problems encountered in attempting to show an association between contaminant exposure and birth defects. Although a few studies or reviews of particular contaminant classes have touched on these difficulties, they have never before been brought together in one review article.

## 2. Methods

PubMed and MEDLINE were searched during June 2018 using the search terms ‘congenital heart defect’ OR ‘congenital heart disease’ AND ‘contaminant’, ‘environmental pollutant’, ‘smoking’, ‘air pollution’, ‘pesticides’, ‘drinking water’, ‘solvents’, ‘landfill sites’, ‘incinerators’, ‘toxic metals’, ‘persistent organic pollutants’ OR ‘volatile organic compounds’. Referenced studies were also obtained. Studies involving recorded disasters, accidents or chemical spills were excluded, as were animal and in vitro studies and non-English language studies. Due to the numerous problems involved in accurately estimating exposure from external measures, a search was also carried out for ‘congenital heart defects’ AND ‘hair’, ‘blood’, ‘serum’, ‘plasma’, ‘placenta’, ‘cord’, ‘milk’, ‘saliva’, ‘salivary’, ‘urine’ and ‘urinary’, in order to be sure of the inclusion of studies using individual biomarker measurements.

For each category of contaminant, if there exists a sufficiently comprehensive meta-analysis or systematic review published after the year 2000, then only studies published after the meta-analysis or systematic review were considered. If there is no relevant meta-analysis or systematic review of a category of contaminant, then studies published since 1990 were included. 

## 3. Results

The search produced 8 meta-analyses and systematic reviews, 3 non-systematic reviews and 58 further studies, after removing excluded papers. 

### 3.1. Smoking

Cigarette smoke contains polycyclic aromatic hydrocarbons (benzo[a]pyrene), tobacco-specific nitrosamines (NNK, NNN), aldehydes (acrolein, formaldehyde), carbon monoxide, hydrogen cyanide, nitrogen oxides, benzene, toluene, phenols (including cresol), aromatic amines (nicotine, 4-Aminobiphenyl), harmala alkaloids and cadmium and may also contain polonium-210. Many of these are known carcinogens [10]. Carbon monoxide, in particular, is able to rapidly cross the placenta and is detectable in fetal circulation at higher levels than in the mother and reduces the availability of oxygen and essential nutrients to fetal tissues by binding to haemoglobin [11]. Cigarette smoke is known to be one of the worst environmental contaminants for many aspects of health. 

Three relatively recent meta-analyses and systematic reviews have shown broadly similar results, with an OR of around 1.1 for maternal smoking and all CHDs, as shown in Table 1. The reason for the relatively low ORs is likely because they are the result of pooling a number of studies showing high ORs with those showing no association or an inverse association. A 2011 systematic review by Hackshaw et al. investigated maternal smoking and any birth defect. Among 19 studies of CHDs, some decades old, they found a pooled OR of 1.09 (25% CI: 1.02, 1.17) for increased risk of any CHD, with only five studies showing a significant association. Analysis of studies which had carried out a one year follow-up showed a slightly increased pooled risk. Septal defects appeared to be the most common finding, but the review did not evaluate the effect of maternal smoking on specific CHD sub-types and did not attempt dose response relationships or any sensitivity analysis [12].

A 2013 meta-analysis of 19 studies by Lee et al. also reported mixed results with a pooled RR of 1.11 (95% CI: 1.02, 1.21) for CHDs among the offspring of mothers who smoked during pregnancy, with a positive association for 12 out of 17 sub-types, the highest RR being 1.44 (95% CI: 1.16, 1.79) for any type of septal defect. Lee et al. had attempted to quantify the likely dose response effect but found no significant association for CHDs overall, compared with women who did not smoke during pregnancy, although there were associations between increasing levels of maternal smoking and increasing risk of septal defects; the dose-response relationship was non-monotonic for other CHD sub-types. The authors also note heterogeneity among studies for any CHD, but not among sub-types, while many were hampered by a small number of cases, differences in classifying smoking status and failure to control for confounding factors. There was also a tendency to group CHD sub-types in order to increase statistical power, which prevented associations being found with specific defects [13].

Zhang et al.’s 2017 meta-analysis results were pooled from 23 studies, only eight of which showed a significant association of maternal smoking with any CHD; the pooled RR was 1.11 (95% CI: 1.04, 1.18). The authors noted considerable heterogeneity among studies, with a few having extreme results and wide confidence intervals. With respect to sub-types of CHD, there was an RR of 1.24 (CI: 1.00, 1.54) for transposition of the great arteries and 1.21 (CI: 1.01, 1.46) for any septal defect, but there was no significant association for other defects. The authors found that in the nine studies assessing a dose-response effect, the pooled RR for any CHD was not significant, although there was a significant correlation for any type of septal defect up to a dose of 15 cigarettes per day, after which the association lost significance as it became non-monotonic; the authors suggest that this may indicate that the pregnancy had ended in still birth or miscarriage. Zhang et al. also point out several weaknesses in the available studies, which can bias the study toward the null hypothesis. For example, although it is known that genetics play a role in CHD incidence, only one study had adjusted for this in logistic regression; although CHDs might result from an effect of passive as well as active smoking, only one study had adjusted for paternal smoking during pregnancy. Additionally, although one study reported that >50% of pregnant smoking women could change smoking intensity many times during pregnancy, the studies generally relied on one smoking assessment during the pregnancy, where it was treated as a single dichotomous variable. Finally, although smoking is a known risk for miscarriage, none of the studies adjusted for this possibility [14].

Since publication of these papers, a 2015 Canadian study found that self-reported active maternal smoking during pregnancy in 1646 subjects, relative to a non-smoking control group of >19,000 subjects, was firstly an independent predictor of fetal death and pre-term delivery and was associated with neonatal death, lower birth weight and other adverse outcomes, as well as being a predictor of all risk factors for several further health conditions, even among those mothers who smoked no more than five cigarettes a day. Not only were their offspring significantly more likely to be born with a cardiac malformation but fetal or neonatal death was often due to such abnormalities [15]. In a 2015 US case control study of >14,000 patients, maternal smoking in the first trimester was independently and dose-dependently associated with CHDs, particularly pulmonary valve and artery anomalies and atrial septal defects; the risks were greater among mothers aged >34 [16]. Meanwhile, a Danish study found that although the risk of individual congenital defects was not increased with maternal smoking, there was a significant increase in multiple defects, including cardiovascular [17], while a 2018 case control study, however, investigated a number of potential risk factors for CHDs and found no significant association with maternal smoking during the first 45 days of gestation [18]. 

Surprisingly, these later studies showing an association with maternal smoking do not have markedly higher ORs for all CHDs; in fact, higher ORs are seen for paternal or passive smoking. A US study found that maternal exposure to second-hand smoke in both the home and workplace was associated with atrial septal defects with an OR of 1.37 (95% CI: 1.09, 1.72) [19], while both maternal and paternal exposure provided an additive effect with an OR of 4.5 (95% CI: 2.5, 8.3), which was increased in offspring who carried the combined null genotype for glutathione S-transferase (GST) GSTM1 and GSTT1 enzymes [20] (genetic polymorphisms are further discussed in Potential Mechanisms of Effect). Paternal smoking of >14 cigarettes a day more than doubled the risk for any CHD, giving an OR of 2.1 (95% CI: 1.3, 3.5) [22], while light paternal smoking increased the risk of conotruncal heart defects, and higher amounts were associated with septal defects and left ventricular outflow tract obstruction [21].

Although these later studies tend to be of improved quality, this does not explain why the ORs for paternal or passive smoking are higher than for maternal smoking; in fact, it may suggest that mothers are under-reporting their own smoking prevalence or extent but are happy to more accurately report the smoking habits of others. A hospital-based case control study investigating hair nicotine levels, which has the advantage of assessing passive as well as active smoking and does not rely on maternal reporting, found a dose-response effect with increasing ORs for increasing hair nicotine levels for all CHDs, septal defects, conotruncal defects, anomalous pulmonary venous return and left and right sided obstructive defects [23]. 

There may be an interaction with BMI; maternal smoking in those overweight or obese had an adjusted OR of 2.60 (95% CI 1.05, 6.47) for septal defects and 3.58 (95% CI 1.46, 8.79) for outflow tract anomalies, compared to ORs of 1.00 (95% CI: 0.58, 1.70) and 0.90 (95% CI: 0.49, 1.64) respectively for maternal smoking and normal BMI; ORs for these defects were not significant for BMI alone, so it is the combination of smoking and higher BMI which puts the fetus at risk [23]. Similarly, maternal homocysteine levels were significantly higher among smokers with an affected pregnancy, while those who also had the methylenetetrahydrofolate reductase (MTHFR) 677 CC genotype had an OR of 11.8 (95% CI: 2.6, 53.3) for a heart defect in offspring [24]. Others have found an interaction between smoking and maternal excision repair cross-complementation group 1 (ERCC1), ERCC5, poly(ADP-ribose)polymerase 2 (PARP2) genes and the infant o-sialoglycoprotein endopeptidase (OSGEP) gene, making the presence of these polymorphisms more likely to result in CHD when the mother smoked [25]. Few studies take these potential interactions into account, suggesting that the risk among certain population groups may be considerably understated. 

Worryingly, the risk for infant mortality exists even for those mothers who were heavy smokers but had stopped smoking during the pregnancy [26]. It is not just the maternal smoker who puts the fetus at risk: A 2011 meta-analysis found that exposure to second-hand smoke significantly increased the risk of stillbirth and total congenital malformations [27]. Even a non-smoking mother with two parents who smoked during her childhood had increased odds of spontaneous abortion [28]. Finally, there is a tendency to regard smoking as a ‘behavior’, forgetting that it is the individual chemicals or mixture of chemicals released in cigarette smoke that is the toxic element. These chemicals are also prevalent in the environment from other sources and may be equally damaging when encountered in the atmosphere. They have all been shown to be teratogenic in animals and to be associated with increased risk of congenital defects [29]. Some of these are discussed in the next section. 

### 3.2. External Air Pollution

The studies of external air pollution mainly cover road traffic exhaust fumes, comprising principally nitrogen dioxide (NO_2_), sulphur dioxide (SO_2_), carbon monoxide (CO), ozone (O_3_), particulate matter 10 (PM_10_) or smaller (≤10 µm); less commonly assessed are other nitrogen oxides, polycyclic aromatic hydrocarbons (PAH) and black smoke. Exposure assessments are generally taken from the routine monitoring of pollutant concentrations at the fixed-site monitoring stations closest to maternal residence at the time of the birth.

Recently, there has been a steep increase in the number of air pollution studies testing an association with congenital anomalies; those with apparently linear results are shown in Table 2 but all are discussed below. A 2011 meta-analysis of 10 studies examining the range of CHDs found that both NO_2_ and SO_2_ were associated with risk of coarctation of the aorta and tetralogy of Fallot and PM_10_ was associated with increased risk of atrial septal defects; other pollutants were not associated with CHDs [30]. The authors refer to the several inverse associations observed in some studies, which will likely have reduced the pooled ORs, although this is not discussed further. They also noted considerable heterogeneity with respect to the diagnostic coding systems, congenital anomaly grouping methods and case definitions among the studies. Distance between maternal residence and monitoring station could vary considerably, as could the number and density of monitors. Exposure measurements were usually then averaged over gestation weeks 3–8, although in one study the average measurements for a year were used, rather than the presumed pregnancy time window. 

A 2014 meta-analysis of 13 studies by Chen et al. [1] found that NO_2_ was significantly associated with coarctation of the aorta with an OR of 1.2 (95% CI: 1.02, 1.41) but associations with other pollutants and other CHDs were not significant. The authors noted several differences in methodology between studies, as well as differences in classification of defect, which made direct comparison unreliable. Chen et al. [1] also noted the frequent lack of adjustment for confounders, such as smoking, maternal age, socio-economic status, parental occupation and season of conception. Although Vrijheid et al. found further associations with specific CHDs [30], Chen et al. had not meta-analyzed a number of these combinations as the number of studies was below their analysis threshold of four studies. They had further carried out two meta-analyses based on whether the variable was continuous or categorical, whereas Vrijheid et al. [30] had converted the continuous variables to categorical in order to increase their statistical power. Although the several studies with inverse associations are shown on Chen et al.’s [1] forest plots, the authors do not comment on them.

A 2017 systematic review of seven purely Chinese studies, including those in the Chinese language in order to overcome potential publication bias, investigated the association of congenital anomalies with exposure to only NO_2_, SO_2_ and PM_10_, as the Chinese government did not monitor other pollutants. Although there were consistent associations of both NO_2_ and PM_10_ with individual CHDs, Jacobs et al. found the clearest correlation between PM_10_ and total CHDs [31]. Again, the existence of inverse associations is not discussed. 

Since these meta-analyses, two large Chinese studies confirmed that PM_10_ exposure of ≥92 μg m^−3^ during the most vulnerable period (3–8 weeks), but not at other gestation periods, was non-linearly associated with congenital heart malformations, including atrial and ventricular septal defects, patent ductus arteriosus and tetralogy of Fallot [32,33]. Another Chinese study found that PM_2.5_ may be a greater risk factor than PM_10_ for ventricular septal defect in the first 10 weeks of pregnancy [34], while a US study showed that higher levels of PM_2.5_ were associated with higher risk of truncus arteriosus, anomalous pulmonary venous return, coarctation of the aorta, interrupted aortic arch and any critical CHD [3]. In the US National Birth Defects Prevention Study, exposure was assessed during pregnancy weeks 2–8 and a positive association was found between NO_2_ and coarctation of the aorta and pulmonary valve stenosis, while exposure to PM_10_ or smaller was positively associated with hypoplastic left heart syndrome but inversely associated with atrial septal defects; these associations were clear when examining individual exposure-weeks, but not when taking a seven week average [35]. 

Studies examining other air pollutants have found that black smoke is associated with malformations of cardiac chambers in the fourth quartile only, with no evidence of a dose response relationship [36], while traffic density studies have also shown that higher levels may be associated with atrial and ventricular septal defects and pulmonary atresia, although the association was not always monotonic [37,38]. Other curious dose response relationships were seen with maternal exposure to NO_2_ and left ventricular outflow tract obstructions and exposure to PM_10_ and hypoplastic left heart syndrome, which were associated at lower exposures but not at higher levels [35]. A Chinese study found a correlation between exposure to O_3,_ assessed during gestational months 1–3, and various CHDs but the risk increased as month of pregnancy increased [34]. A US study found that NO_2_ exposure was positively associated with ventricular septal defects but the risk could be reduced with higher intake of methyl donor micronutrients [39]. Zhang et al. noted that a UK study had found an association between cardiac defects and SO_2_ levels, whereas a Chinese study found no association, despite considerably higher SO_2_ levels in China compared to the UK [34]. 

### 3.3. Pesticides

The category of ‘pesticides’ is usually taken to comprise insecticides, rodenticides, fungicides, herbicides and may even include nitrogen fertilizers; these represent a wide range of chemicals with different mechanisms of effect, although some are known to act as endocrine disruptors. Usually the proximity of the birth residence to crop spraying is taken as the exposure of interest. Some human studies have found that pesticide exposure shows correlations to decreased fertility as well as intrauterine growth retardation, preterm birth and low birth weight [40]. There is good reason to suspect that maternal pesticide exposure affects the fetus or infant, since detectable levels have been found in maternal breast milk, amniotic fluid and cord blood [41,42], and birth defects have been found in exposed animals [42]. 

No meta-analyses have been carried out, although a 2011 review of 25 studies of adverse reproductive outcomes and proximity to agricultural pesticide applications reported that only one study from 1981 had considered congenital heart defects and that had found no association [40]. One reason for this may be that earlier studies considered broad categories of exposure and none considered a correlation between a specific chemical and a CHD [43]. Since the 2011 review, however, there have been larger studies of higher quality, as shown in Table 3. A 2016 case control study of >300,000 infants investigated maternal agricultural pesticide exposure based on application to crops within 500 m of maternal residence at birth as a surrogate for exposure at gestational ages 20–44 weeks. Among the >6000 infants with birth defects, there was a significant association with total birth defects with an OR of 1.98 (95% CI: 0.69, 5.66), and specifically for atrial septal defects with an OR of 1.70 (95% CI: 1.34, 2.14) and patent ductus arteriosus with an OR of 1.50 (95% CI: 1.22, 1.85) at the highest level of exposure; mothers with diabetes had a higher risk of having an infant with a birth defect [41]. The second case control study investigated exposure to commercial pesticide application within a similar radius of the mother’s address during a three month periconceptional window and found that pregnant women could have come into contact with up to 53 groups of chemicals and 248 individual chemicals. There were significant associations for individual insecticide and fungicide categories and tetralogy of Fallot, hypoplastic left heart, coarctation of the aorta, pulmonary valve stenosis and ventricular and atrial septal defects, but because they specified individual pesticides, their results cannot be compared to those in other studies [43]. 

To indicate the need to consider greater pesticide exposure than merely crop spraying, a case control study of exposure to domestic pesticides, either applied by the mother or a professional, showed that exposure was associated with conotruncal defects, the only CHD investigated in this particular study [44]. Another study investigating any known type of exposure, domestic or agricultural, found an association with incidence of transposition of the great arteries, with OR 2.0 (95% CI 1.2, 3.3), although no relationship was found with other heart defects; specific correlations were seen with herbicides, with OR 2.8 (95% CI: 1.3, 7.2) and rodenticides with OR 4.7 (95% CI: 1.4, 12.1) but the relationship was broadly similar whether there was a single or continuous exposure [42]. Higher maternal occupational insecticide exposure from one month prior to conception to the end of the first trimester was found in the National Birth Defects Prevention to be associated with atrial septal defects, while higher exposure to both insecticides and herbicides was associated with hypoplastic left heart syndrome, pulmonary valve stenosis and tetralogy of Fallot compared to controls; fungicides were also associated with tetralogy of Fallot [45].

### 3.4. Contamination of the Household Water Supply

The most widely investigated contaminants of the public water supply are disinfectant by-products (DBPs), which can be formed as part of the disinfection (mainly chlorination) of water when organic matter in the water reacts with the disinfectant. Around 600 DBPs have so far been identified and their formation and occurrence depend on many factors. They may exist singly or in mixtures, making it difficult to assess individual DBP health effects, particularly in epidemiologic studies. Drinking water is thought to be the main route of exposure for non-volatile DBPs, while exposure to volatile DBPs, such as the trihalomethanes (THMs) chloroform, bromodichloromethane, dibromochloromethane and bromoform, can also occur through inhalation and absorption during activities such as showering and bathing, although swimming appears to generate the highest level of THM uptake in the blood. Other potential contaminants include pesticides, toxic metals and fluoride [46].

A 2009 meta-analysis (Table 4) found significantly higher risk of 17% for total congenital anomalies with exposure to water chlorination or trihalomethanes but this was based on a small number of studies and there were several which showed no association. There was also a 58% increased risk for ventricular septal defects, but this was based on only three studies and the relationship was not monotonic. The authors make the point that the majority of studies investigated trihalomethanes as a surrogate for total DBP burden, without considering other DBPs such as haloacetic acids which may have varying effects and whose metabolism is completely different. Exposure to DBPs in conjunction with low concentrations of methyl donors in the form of folate were associated with several congenital defects [46].

A study investigating maternal exposure through swimming, showering and bathing found little association with CHD for total trihalomethanes or chloroform but there was an association with bromodichloromethane, which had a dose-dependent >2-fold increased risk during the first month of pregnancy and an increased risk overall in the first trimester [47]. There have been no subsequent studies. 

### 3.5. Solvents

Solvents are usually found as liquids but can also take the form of solids or gases, depending upon temperature and other conditions. Volatile solvents are regularly found in dry cleaning fluid (as tetrachloroethylene, and previously as trichloroethylene), spot remover, paint thinner, nail varnish remover, perfume and glue. Trichloroethylene, a halogenated hydrocarbon, is now primarily used as a metal degreasing agent but has also been used as an anesthetic, as an inhaled obstetric analgesic and in coffee decaffeination; it is known to contaminate drinking water supplies [48]. 

As shown in Table 5, a 2006 review concluded that results of both human and animal studies linking exposure to trichloroethylene with congenital heart defects are inconsistent and interpretation has been found difficult; the authors found no association for any specific heart defect [48]. A similar conclusion was drawn in a 2005 review of the effects of trichloroethylene and dichloroethylene [49]. Nevertheless, since then the 2012 National Birth Defects Prevention Study, which assessed the impact of solvent exposure from one month before conception to the end of the first trimester, found significant associations for exposure to any solvent, with OR 1.6 (95% CI: 1.0, 2.6), and for association of any chlorinated solvent, with OR 1.7 (95% CI: 1.0, 2.8), with ventricular septal defects. An alternative approach to assessing solvent exposure found stronger associations between any solvent exposure and aortic stenosis and Stoddard solvent exposure and transposition of the great arteries, right ventricular outflow tract obstruction and pulmonary valve stenosis, with ORs exceeding 2 in some cases [50].

### 3.6. Toxic Metals

Metals, primarily comprising arsenic, cadmium, manganese and lead, are known human developmental toxicants that are able to cross the placental barrier. There are no meta-analyses or systematic reviews of toxic metal exposure and CHDs, and many of the individual studies, often non-English language, involve exposure to arsenic. This occurs naturally in drinking water in several parts of the world, and may be deposited from metal mining and smelting or from use as an agricultural pesticide. While older studies test the metal concentration of drinking water, more recent studies have tested hair concentrations. 

Studies using drinking water or other environmental exposure are shown in Table 6a; those using maternal hair or placental levels are shown in Table 6b. An early drinking water study had found an association of arsenic with coarctation of the aorta, with OR 3.4 (95% CI: 1.3, 8.9) but not with any other CHD and no association at all for lead or mercury; the presence of selenium proved to have a dose-responsive inverse association, suggesting that it may be protective [51]. A more recent Hungarian case control study investigated nearly 10,000 cases of congenital heart defects and found an association between an arsenic level >10 μg/L (the current EU limit) and total CHD incidence, with OR 1.41 (95% CI: 1.28, 1.56), with the risk of ductus Botalli persistens and atrial septal defects having ORs of 1.81 (95% CI: 1.54, 2.11) and 1.79 (95% CI: 1.59, 2.01) respectively [52]. A study from Bangladesh showed that drinking water arsenic was associated with birth defects in general [53], while well water manganese was associated with a higher prevalence of conotruncal heart defects, although there was no association with arsenic, cadmium and lead [54]. 

When metal concentrations were measured in hair, it was found that arsenic levels were significantly higher in almost every CHD sub-type and were dose-dependent [55]. Hair cadmium was also associated with a 2.81-fold increase in the incidence of conotruncal defects for levels in the highest group and there was an additive effect of the presence of both cadmium and arsenic [55]. Hair lead was also found to be dose dependently associated with the presence of certain sub-types of congenital heart defects in a Chinese case control study [56]. Additionally, hair copper was associated with total CHDs, but particularly conotruncal defects [57], hair aluminum was associated with total CHDs and particularly septal and conotruncal defects and right ventricular outflow tract obstruction [58], and hair barium was associated with total CHDs and particularly with septal defects, conotruncal defects, right and left ventricular outflow tract obstruction and anomalous pulmonary venous return [59]. Only one study has assessed maternal blood metals and found that high blood lead was associated with total CHDs, conotruncal defects, septal defects and right ventricular outflow tract obstruction, while higher blood selenium was protective; cadmium, chromium, copper and mercury were not associated their own but may have had an interactive effect [60]. 

Other studies have shown particular problems with lead. An area of Italy polluted with lead from ceramic factories was found to have a higher incidence of cardiovascular defects, which decreased with time after the pollution was caused [61], while an investigation of paternal employment in a US microelectronics/business machine manufacturing facility found that lead exposure was associated with increased risk of ventricular septal defect, with OR 2.7 (95% CI: 1.09, 6.67) [62]. A recent study investigated cord blood and found that presence of aluminum was associated with higher incidence of CHDs, with OR 2.08 (95% CI 1.11–3.88) [63]. 

### 3.7. Landfill Sites and Incinerators 

Routes of exposure to the chemicals emitted by landfill sites or incinerators include airborne, household waterborne and contaminated soil; even the toxic emissions from crematoriums may deposit on soil or in rivers. Incinerators may also emit most of the chemicals reviewed under external air pollution, with the addition of hydrogen chloride acid (HCl), cadmium, lead, mercury, chromium, arsenic and the persistent organic pollutants dioxins, furans, polychlorinated biphenyls (PCBs), polycyclic aromatic hydrocarbons (PAHs) and radioactive wastes. Modern incinerators have much improved emission control but are expensive and few countries have decided to invest in them for the health of their populations. Furthermore, improved emission control means a higher quantity of highly toxic fly ash remains, containing mainly heavy metals and dioxins, which is generally then placed in hazardous waste sites. 

Table 7 shows the principal studies involving landfill sites and incinerators. A 2014 systematic review of maternal proximity to incinerators noted a high degree of heterogeneity between studies in design, quality, location, time period, number of incinerators and the health outcome investigated; nevertheless, those studies deemed to be of higher quality showed a significant association with fatal heart defects, as well as a number of other anomalies [64]. There are more studies investigating proximity to landfill or hazardous waste sites. A large 2009 US case control study found that living within a mile of any waste site was associated with truncus arteriosus, with OR 2.8 (95% CI 1.19, 6.54) but living within a mile of a hazardous waste site gave an OR of 4.63 (95% CI: 1.18, 13.15), but there was no association with risk of conotruncal heart defects as a whole [65]. Earlier studies had found no associations with non-fatal defects [66] but there were associations with congenital heart and circulatory system defects, including transposition of the great arteries [67,68,69]. A five-country EUROHAZCON study found that maternal residence within 3 km of a landfill site was associated with a significantly increased risk of total congenital anomalies and specific defects of the septum, great arteries and veins; the association decreased with residential distance away from the site. The study ranged from uncontrolled waste sites to relatively modern controlled operations, so that it makes it almost impossible to determine whether the risk was linked to one specific type of site or chemical; there was similarly no indication that risk of congenital defect was associated with the ranking of the hazard potential of the sites [70]. 

### 3.8. Persistent Organic Pollutants and Other Contaminants

As shown in Table 8, there are also a few isolated studies of persistent organic pollutants, particularly phthalates, which are used as plasticizers in the manufacture of polyvinylchloride (PVC) flooring products, adhesives, detergents, toiletries, vehicle interiors, medical tubes and containers and have been found in many foods, possibly from plastic containers. A Han Chinese study found that maternal occupational exposure to phthalates and alkylphenolic compounds were associated with a higher incidence of total congenital heart defects, with ORs 1.6 (95% CI: 1.0, 2.6) and 1.8 (95% CI: 1.1, 3.0) respectively, but it was found that infants carrying the ABCB1 gene C3435T polymorphism were at greater risk, particularly for septal defects [71]. A later Chinese study confirmed the risk of maternal occupation exposure to phthalates, alkylphenolic compounds and heavy metals as risk factors for ventricular septal defect, atrial septal defect, patent ductus arteriosus and pulmonary valve stenosis, while paternal exposure to phthalates or alkylphenolic compounds was a risk factor for ventricular septal defect and pulmonary valve stenosis [72]. Another Chinese study found an association between all CHDs and maternal housing renovation exposure, with OR 1.89 (95% CI: 1.29, 2.77) and if the mother had moved into a new house shortly after decoration within three months prior to or after conception, with ORs 2.38 (95% CI: 1.03, 5.48) and 4.00 (95% CI: 1.62, 9.86), respectively, particularly conotrucal defect and anomalous venous return [73]. 

Two European studies found that although maternal occupational phthalate exposure was not associated with congenital heart defects, paternal exposure to phthalates and polychlorinated compounds carried a higher risk for ventricular septal defects, while exposure to alkylphenolic compounds was associated with coarctation of the aorta [74,75]. Similarly, a US study considered paternal occupational exposure in the six months prior to conception and found an association between septal defects and jewelry-making, welding or ionizing radiation and endocardial cushion defect with Down’s syndrome, lead soldering and pulmonary atresia and paint stripping and hypoplastic left heart in infants with a family history of cardiac defects [76]. Parental exposure to hair dye and laboratory chemicals was also found to be a risk factor [69]. 

## 4. Potential Mechanisms of Effect

From human and animal research, several potential mechanisms of effect of environmental contaminants in the development of birth defects have been proposed. These comprise the indicators of individual susceptibility, such as increased reactive oxygen species (ROS), reduced mitochondrial energy production, increased inflammatory cytokines, upregulation of the aryl hydrocarbon receptor, reduced maternal folate intake and embryonic hypoxia [30,31,41,47,77]. Studies have noted that in utero nicotine exposure could induce fetal hypoxia, decrease heart rate and raise blood pressure [78], while embryonic hypoxia has been shown to induce several types of CHD, mediated by the unfolded protein response (UPR), which reduces fibroblast growth factor signaling in cardiac progenitor cells [79]. Many environmental contaminants can induce embryonic UPRs [79]. 

There is also some support for aryl hydrocarbon receptor upregulation, since it is known to regulate expression of genes critical in controlling cardiogenesis. Genetic ablation or blocking of the receptor during early development was shown to cause structural and functional cardiac abnormalities, as well as degraded mitochondrial oxidative phosphorylation [80], although another study indicated that genetic ablation could be protective [77]. Elevated homocysteine concentrations have already been mentioned, but several studies have noted that both reduced folate intake and elevated homocysteine can be confounding factors suggestive of impaired methylation of histones and DNA [81,82,83,84]. This is supported by a Canadian study which found that folic acid food fortification could reduce the prevalence of several types of congenital heart disease [85]. 

Several of the studies discussed in this paper investigated genetic polymorphisms which might affect the impact of environmental contaminants on CHDs. Most often tested is the MTHFR 677 CC genotype. Since smoking is known to raise homocysteine and individuals with this genotype also tend to have elevated homocysteine levels, the combination is likely to markedly increase the risk of birth defects. One study showed that the risk of CHD was almost 12 times higher in mothers with the polymorphism who also smoked, compared to non-smoking mothers without the genotype [27]. GST genotypes were also investigated in smokers with findings that the combined null genotype for GSTM1 and GSTT1 enzymes produced an additive effect for atrial septal defects when combined with parental exposure to second-hand smoke [23]. The GST genes are a family of detoxification enzymes, which are critical for defense against environmental contaminants, with earlier studies showing that deletion of these genes could contribute to several congenital defects. The combined null genotype therefore suggests a particular problem with metabolizing contaminants [22]. Others have found an interaction between smoking and maternal excision repair cross-complementation group 1 (ERCC1), ERCC5, poly(ADP-ribose)polymerase 2 (PARP2) genes and the infant o-sialoglycoprotein endopeptidase (OSGEP) gene, making the presence of these polymorphisms more likely to result in CHD when the mother also smoked [28]. These genes are principally involved in DNA repair pathways and can represent either a protective mechanism with maternal smoking or an increased risk factor for birth defects, depending upon the specific polymorphism involved, although few studies have investigated these and the precise mechanisms remain unclear [28]. 

Finally, a Han Chinese study found that although maternal occupational exposure to various persistent organic pollutants was associated with a higher incidence of CHDs, infants carrying the ABCB1 gene C3435T polymorphism were at greater risk, particularly for septal defects [71]. This gene regulates expression of placental ATP-binding cassette (ABC) toxicant transporters and hence plays a crucial role in teratogenic protection against maternal contaminants; ABCB1 knock-out mice have shown fetal birth defects when exposed to certain drugs, while the C3435Y single nucleotide polymorphism is associated with increased toxicant transport across the placenta. 

## 5. Discussion

### 5.1. Findings from the Review

The findings from meta-analyses, systematic reviews and more recent investigations indicate that while many studies show an association with any CHD or a specific CHD, there are also a number which show no association; this means that any pooled risk estimate is fairly modest. To some extent, those studies showing no association tend to be older and of poorer design, while those that show an association tend to be more recent, with a considerably larger population and are of better quality. However, just as there is no contaminant or contaminant group which consistently shows a clear association with CHDs, there is similarly no contaminant or contaminant group which consistently shows no association with CHDs. Similarly, although septal defects are possibly the most common defect for which an association can be seen, there is no specific defect which is associated with any particular contaminant. This was starkly highlighted by the UK study which had found an association between cardiac defects and SO_2_ levels, whereas a Chinese study found no association, despite considerably higher SO_2_ levels in China compared to the UK.

Despite the limited consistency of results, meta-analyses nevertheless show a modest association between smoking and all CHDs and with septal defects and transposition of the great arteries, with a similar association of NO_2_ with coarctation of the aorta; SO_2_, PM_10_ and PM_2.5_ may also be associated with CHDs. A meta-analysis of drinking water trihalomethane and chlorination by-product exposure similarly found a modest association for total congenital defects, with greater correlation for septal defects; a study of exposure through bathing and showering found an association with bromodichloromethanes. A systematic review similarly found a small but significant association between residential proximity to incinerators and congenital anomalies and fatal heart defects. Although there are no meta-analyses regarding agricultural pesticide exposure, large US studies have shown associations with total birth defects, with insecticide and fungicide exposure correlating with specific CHDs, while the single study of domestic pesticide exposure also showed an association. There are similarly no meta-analyses or systematic reviews of the effect of exposure to solvents, a range of metals, landfill sites or hazardous waste sites, yet the relatively few recent studies again tend to show an association with CHDs.

Several studies have attempted to demonstrate a dose response effect, yet many have failed to do so. For example, there is a dose response effect between smoking and septal defects, but only up to 15 cigarettes per day, after which the association becomes non-monotonic, possibly because at higher doses the pregnancy has ended in miscarriage. This reflects a particular weakness of many of these studies that fail to account for miscarriage, abortion or stillbirth due to congenital heart defects; as a result, the prevalence of CHD is not included in the study, so that the full extent of exposure and condition are not recorded. This may be the reason for some of the apparent inverse effects of certain contaminants, i.e., they account for a disproportionately high number of fetal deaths or stillbirths, so that it appears as if the control population has a higher number of live congenital defects. External air pollution studies have shown a particularly high number of inverse associations; it may be that the nature of air pollution is so damaging that fewer of the pregnancies result in live births. 

Similarly, the association between exposure to several air pollutants and CHDs was found to be non-linear, with some showing a relationship at lower doses which was absent or inverse at higher doses [3,86]. Interestingly, a dose response relationship was evident with respect to hair metal levels and CHDs, possibly indicating that a personalized measurement is more accurate than extrapolating from an estimated proximity measure. It could also reflect the fact that many of these contaminants are endocrine disruptors, which are known to have non-monotonic dose response relationships, where there may be a greater health effect at low dose than at high dose. Although particular care was taken to search for studies using personal biomarkers, only a few were found for hair measurements and only one for blood. Other than a hair nicotine study, all the remainder involved metals and showed consistent associations for arsenic, although there were insufficient numbers of studies to draw conclusions about other metals. Greater use could be made of personal biomarkers, using not just maternal hair and blood but also placenta, cord blood, saliva and urine, as well as adipose tissue to reflect older exposures. 

### 5.2. Potential Mechanisms

Among the few studies investigating mechanisms of effect of environmental contaminants and CHDs, the majority tend to focus on fetal hypoxia, upregulation of the aryl hydrocarbon receptor and elevated homocysteine. Excess homocysteine is also a feature of the MTHFR 677 CC genotype, with the incidence of CHDs being exacerbated by maternal smoking. Polymorphisms in the antioxidant GST genes, the placental transport ABCB1 gene, as well as various DNA repair genes can also have an additive effect with environmental contaminants on CHD incidence. 

These studies suggest that the effect of genetic polymorphisms on incidence of CHDs may be exacerbated by the presence of environmental contaminants. For example, genetic variants in transforming growth factor beta (TGF-β) receptors type 1 (TGFBR1) and type 2 (TGFBR2) genes are commonly found in Marfan syndrome and can also be associated with patent ductus arteriosus and VSDs. Although none of these gene studies have considered the effect of maternal environmental contaminant exposure, this highlights the need to carry out high quality studies based on personalized genetic analysis together with individual environmental contaminant exposure. 

### 5.3. Methodological Problems 

Many of the earlier studies fail to adjust for potential confounders, and even some of the more recent studies do not consider an adequate range. Several papers have noted that risks rise with maternal age, but few correct for BMI, presence of type 2 diabetes, occupation or socio-economic status, which are also known risk factors. Similarly, few take maternal or fetal genetic polymorphisms, maternal homocysteine concentrations or folate intake into consideration, or adjust for maternal or paternal smoking, despite this being among the clearest of the associations. In addition, the issue of preconception paternal exposure is rarely considered, yet in studies where it has been taken into account (principally smoking but also exposure to metals and persistent organic pollutants), it is almost always associated with prevalence of CHDs 

The authors of all the meta-analyses and reviews note the considerable heterogeneity between studies, making an accurate comparison extremely difficult. There are also a number of inherent assumptions which may prove inaccurate. For example, many studies only investigate contaminant exposure during weeks 3–8, although at week 8 cardiac development is not complete and may yet be compromised; one study found that that the severity of birth defects increased as pregnancy advanced beyond the 8 weeks. Furthermore, where the last date of menstruation is unclear, the potential exposure time may be misstated. Moreover, measurement of exposure during the first trimester assumes no impact on the fetus from maternal or paternal pre-conception exposure. Yet the more dangerous lipophilic pollutants can be stored in the body, usually in adipose tissue, and may be released with exercise and weight loss regimens, and can affect sperm, ova or the growing fetus at any time. Finally, the birth location is assumed to be the maternal residence during the critical weeks in the first trimester, although the mother may have moved house during her pregnancy; earlier studies have indicated that up to 33% of females move house during the pregnancy [87]. 

Considerable differences have been seen in the results between using one exposure assessment during the pregnancy, taking an average of several, or considering exposure weeks individually. Similarly, exposures can be taken as either dichotomous or continuous variables to include exposure extent; two meta-analyses of the same contaminant treated these variables differently and obtained different results. The grouping of CHD types can increase statistical power but loses the associations seen by assessing CHDs individually; studies also varied in diagnosing, defining and classifying anomalies. In addition, a heart defect may not be diagnosed until after the study period is complete, normally a few weeks after birth; many are not diagnosed until later life. 

The frequent grouping of types of chemical, such as ‘pesticides’, fails to identify a particular problem with, for example, organochlorine herbicides, which are not seen in pyrethroid insecticides as they have a different mechanism of action. There may also be lack of standardization in mixtures and concentrations of pesticide sprays and water disinfectant chemicals or differences in smoking status (i.e., ever smoking, current smoking, pack years etc.). With respect to pesticides, air pollution, landfill sites and incinerators, there is often a large difference in the cut-off distances between maternal residence and monitoring station between studies, with little adjustment for distance from source. Additionally, the average of the daily pollutant measurement is sometimes used, whereas other studies take the daily maximum concentrations, arguing that these would better reflect any harmful exposure and more closely align with regulatory standards. With this type of exposure assessment there is an assumption of homogeneity of exposure in a similar radius around the measuring station or application point, but this fails to take into account wind strength and direction and other weather events, and any spatial distribution pattern for individual pollutants, which would all have a bearing on the extent of exposure. Similarly, pregnant women who spend more time indoors in a cold climate may be less exposed than those spending more time outdoors or with doors and windows habitually open. Duration of exposure is also rarely considered. There is no coherent monitoring plan for licensed agricultural, domestic and industrial products and even less awareness of illegal practices. 

Other problems noted by a number of authors include the limited definitions of exposure. For example, air pollution studies may measure the vehicle exhaust chemicals at a monitoring station, while ignoring occupational or industrial emissions, while pesticide application is generally taken as crop spraying, ignoring the routine spraying of public spaces such as shopping centers, schools and hospitals, any domestic application, which often ends up in highly toxic household dust, and pesticide content of non-organic foods and the water supply. In a US agricultural area, the herbicide atrazine was found in around 8% of public water systems and its degradation product desethylatrazine was found in 10–12% of water samples [88]. Likewise, most studies consider water only from the perspective of drinking and do not assess extent of swimming, showering or bathing, yet many DBPs are volatile organic compounds (VOCs) and hence can be inhaled or may be absorbed through the skin. As well as further unquantified exposures to the same chemical, it is also assumed that pregnant women are exposed to no other contaminants whatsoever. This is a completely unrealistic assumption, given the hundreds of contaminants to which everyone is exposed on a daily basis. Failure to include other potential exposures greatly under-estimates the risks and distorts the study results, as well as failing to take into account any interaction effect. 

This leads to one of the greatest problems encountered in these studies of toxic exposures, namely the use of surrogate measures to estimate actual exposure, with all the attendant problems of measurement and standardization. The estimates are inherently unreliable for all the reasons discussed above, but there is also so much personal metabolic variation that the impact of a fixed exposure dose will differ considerably by individual. This is an issue common to all environmental medicine studies and suggests that a standardized means of personalized measurement, such as cord blood or hair metals, should be developed as a matter of urgency. Finally, there are all the problems previously mentioned concerning the exclusion from the study of fetuses with CHDs which were spontaneously or deliberately aborted or resulted in stillbirth or neonatal death, as well as the diagnosis of a CHD later in life and long after the study has ended. Both these issues will understate the number of cases and consequently the risk. To some extent this can now be overcome by the use of fetal echocardiography and biomarkers, such as pregnancy-associated plasma protein-A and free beta human chorionic gonadotropin, tested during the first or second trimester [89,90,91].

## 6. Conclusions

As is the case with many recent studies of the associations of environmental contaminants and human health, this review has found considerable evidence of a relationship with congenital heart defects. As several earlier studies failed to show an association, either because of study size or poor design/methodology, this means that attempts in meta-analyses to calculate a pooled risk for each contaminant or contaminant class show only a very modest effect, which masks the often much clearer correlations in the later studies. One of the strongest associations found in meta-analyses is for maternal smoking; some of the many components of cigarette smoke are also found in external air pollution, which shows a similarly consistent association for NO_2_, SO_2_, and PM, as well as incinerator emissions. Associations have also been seen in a meta-analysis of disinfectant by-products. Although there are no meta-analyses or systematic reviews, recent studies have shown correlations with pesticides, solvents, metals and landfill/hazardous waste sites. 

From this range of results, it is possible to draw some useful conclusions. Firstly, although septal defects are the most commonly observed, there is no contaminant or contaminant group which is associated with any particular defect. Secondly, there is no clear dose response effect, likely due to severe exposure resulting in miscarriage or stillbirth, thereby distorting results. Other potential reasons include the non-monotonic effect of endocrine disruptors, as well as the known propensity for a larger effect at lower doses. Other common difficulties in studies of this nature include the unreliability of estimates of exposure to contaminants such as pesticides and external air pollution, which rely on distance from the contaminants, without taking into account variables such as weather, likely exposures from other contaminants and the assumption that maternal residence at conception is identical to residence at birth. Additional problems include the failure of studies, particularly earlier studies, to adjust for the range of likely confounders, as well as the assumption that the only exposure likely to have an effect occurs between gestation weeks 3–8. A similar failure to consider maternal and paternal preconception exposure or later life diagnoses of CHDs can understate the associations. Failure to treat exposures appropriately as either dichotomous or continuous, as well as the grouping of contaminant types or CHDs to increase statistical power, fails to detect an effect of specific contaminants on specific defects. 

Since, for ethical reasons, an RCT of environmental contaminants will never be carried out, we must rely on observational studies to assess their risk to the unborn child. A recent review of all birth defects concluded that there is sufficient epidemiological evidence to demonstrate a causal association between maternal smoking and CHDs, although the evidence from studies of other contaminants was limited [92]. This makes it all the more important that case control studies are carried out using early diagnosis of CHDs, such as through fetal ultrasound and biomarkers, and that improved means of assessing individual total exposure is progressed, using personal biomarkers such as hair, blood, urine, adipose tissue and placenta. 

## Figures and Tables

**Table 1:** Maternal smoking and congenital heart disease: Main findings from the literature.

Author Ref.	Article Type	All CHDs or Any CHD Subtypes	Study Limitations
Hackshaw et al. [12]	Systematic review	All CHDs: Pooled OR: 1.09 (25% CI: 1.02, 1.17)	As per Zhang et al.
Lee et al. [13]	Meta-analysis	All CHDs: Pooled RR 1.11 (95% CI: 1.02, 1.21) Any SD: Pooled RR 1.44 (95% CI: 1.16, 1.79)	As per Zhang et al. Some early studies are very small.
Zhang et al. [14]	Meta-analysis	All CHDs: Pooled RR 1.1 (95% CI: 1.04, 1.18), TGA: Pooled RR 1.24 (CI: 1.00, 1.54). Any SD: pooled RR 1.21 (CI: 1.01, 1.46).	Studies have low heterogeneity and generally do not consider effect of genetics, change in smoking volume, all confounding factors, exposure to passive smoking or pregnancy ending in death.
Mei-Dan et al. [15]	Retrospective cohort study	All CHDs: 1.5% vs. 0.8%, *p* < 0.05	Did not consider genetics or many other confounding factors.
Sullivan et al. [16]	Population-based study	All CHDs: OR 1.16 (95% CI: 1.08, 1.24) ASDs: OR 1.22 (25% CI: 1.08, 1.38)	Possible inadequate classification of CHDs; used self-reported smoking status; likely inadequate confounding factors.
Leite et al. [17]	Population-based cohort study	All CHDs: OR 1.13 (95% CI: 1.07, 1.19)	Did not consider genetics or many other confounding factors.
Arimandnia et al. [18]	Case control study	All CHDs: No association for maternal smoking gestation days 1–45	Questionnaire-based study so possible recall bias.
Hoyt et al. [19]	Population-based case control study	ASDs: Second hand smoke exposure ORs: 1.37 (95% CI: 1.09, 1.72)	No data on extent of exposure, so no calculation of dose response effect. Interviews conducted some time after the birth—possibility of recall bias.
Cresci et al. [20]	Case control study	All CHDs: Paternal smoking: OR 2.1 (95% CI: 1.3, 3.5). Second hand smoke exposure: Maternal OR 2.6 (25% CI: 1.6, 4.2); paternal OR 2.5 (25% CI: 1.6, 3.8); combined OR 4.5 (95% CI: 2.5, 8.3).	Smoke exposure self-reported.
Deng et al. [21]	Hospital-based case control study of paternal smoking	Conotruncal defects: OR 2.23 (95% CI: 1.05, 4.73) Septal defects: OR 2.04 (95% CI: 1.05, 3.98) Left ventricular outflow tract obstruction: OR 13.12 (25% CI 2.55, 67.39)	Not representative of population. Possible recall bias. Other maternal exposure not excluded.

Abbreviations: TGA: Transposition of the great arteries; SD: Septal defect; ASD: Atrial septal defect.

**Table 2:** External air pollution and congenital heart disease: Main findings from the literature.

Author Ref.	Article Type	All CHD or Any CHD Subtypes	Study Limitations
Vrijheid et al. [30]	Meta-analysis	NO_2_: COA: OR 1.17 (25% CI 1.00–1.36); TOF: OR 1.2 (25% CI 1.02, 1.42) SO_2_: COA: OR 1.07 (25% CI 1.01, 1.13); TOF: OR 1.03 (25% CI 1.01, 1.05) PM_10_: ASD: OR 1.14 (95% CI 1.01, 1.28)	Studies were few, high heterogeneity, different methods and extent of exposure measurement, different classifications of CHD and diagnostics, assumes a linear dose response relationship, generally do not consider effect of genetics, all confounding factors or pregnancy ending in death.
Chen et al. [1]	Meta-analysis	NO_2_: COA: OR 1.20 (25% CI 1.02, 1.41). Other CHDs and pollutants not significant.	As per Vrijheid et al. Did not meta-analyse where only a few studies.
Ren et al. [32]	Population based study	All CHDs: PM_10_ exposure of ≥92 μg m^−3^ OR 1.16 (95% CI: 1.06, 1.28)	Machine learning study, but insufficient studies of this nature to determine its usefulness.
Liu et al. [33]	Hospital-based case control study	PM_10_ exposure associated with: All CHDs: OR 1.28 (95% CI: 1.03, 1.61) ASDs: ORs 1.29 to 2.17 (95% CI: 1.17, 2.23) VSDs: OR 1:19 (95% CI:1.00, 1.43) PDA: ORs 1.54 to 1.63 (95% CI: 1.06, 3.24) TOF: OR 1:44 (95% CI: 1.01, 2.19)	Uncertainty over exposure monitoring. Pregnancy duration <20 weeks was ignored, leading to bias as a CHD may have been the reason. Early pregnancy residential location was used, which is more representative but makes comparison with other studies using birth location more difficult. Few potentially confounding factors assessed.

Abbreviations: COA: Coarctation of the aorta; TOF: Tetralogy of Fallot; ASD: Atrial septal defect; VSD: Ventricular septal defect; PDA: Patent ductus arteriosus.

**Table 3:** Pesticides and congenital heart disease: Main findings from the literature.

Author Ref.	Article Type	All CHDs or Any CHD Subtypes	Study Limitations
Rappazzo et al. [41]	Case control study	ASD: OR 1.70 (95% CI: 1.34, 2.14) PDA: OR 1.50 (95% CI: 1.22, 1.85)	Use of registry data limits adjustment for confounding factors. Considered only live births. Maternal residence at birth used; some rural addresses excluded, potentially under-reporting exposure.
Loffredo et al. [42]	Population based study	Any pesticides: TGA: OR 2.0 (95% CI 1.2, 3.3) Herbicides: TGA: OR 2.8 (95% CI: 1.3, 7.2) Rodenticides TGA: OR = 4.7 (95% CI: 1.4, 12.1) Insecticides: No association.	Considered only live births. Did not consider full range of confounding factors.
Carmichael et al. [43]	Case control study	TOF: Neonicotinoids: OR 2.4 (95% CI 1.1, 5.1) HLH: Strobin: OR 2.9 (95% CI: 1.2, 7.0); triazine: OR 2.2 (95% CI: 1.0, 5.1) COA: Pyridazinone: OR 2.9 (95% CI 1.1, 7.5) VSD: Avermectin: OR 2.8 (95% CI 1.2, 6.2) ASD: Dichlorophenoxy acid: OR 2.3 (95% CI 1.2, 4.5)	Small sample sizes for individual pesticides. Exposure assessment only involved agricultural pesticides. No assessment of wind and weather conditions or individual metabolism.
Rocheleau et al. [45]	Case control study	All CHDs: No association ASDs: Insecticides: OR 1.8 (95% CI: 1.3, 4.7) HLH: Insecticides and herbicides: OR 5.1 (95% CI: 1.7, 15.3) PVS: Insecticides and herbicides: OR 3.6 (95% CI: 1.3, 10.1) TOF: Insecticides, herbicides and fungicides: OR 2.2 (95% CI: 1.2, 4.0)	Could only investigate pesticide categories, yet individual pesticides may have a different effect to the overall category. Not all potential confounders could be included.

Abbreviations: HLH: Hypoplastic left heart; PVS: Pulmonary valve stenosis; COA: Coarctation of the aorta; TOF: Tetralogy of Fallot; ASD: Atrial septal defect; VSD: Ventricular septal defect; PDA: Patent ductus arteriosus; TGA: Transposition of the great arteries.

**Table 4:** Contamination of the household water supply and congenital heart disease: Main findings from the literature.

Author Ref.	Article Type	All CHDs or Any CHD Subtypes	Study Limitations
Nieuwenhuijsen et al. [46]	Meta-analysis	Trihalomethanes: All CHDs: OR 17% (95% CI: 3, 34) VSDs: OR 58% (95% CI: 21, 107)	Small number of studies. Differences in exposure measurement and cut-off points.
Grazuleviciene et al. [47]	Cohort study	All CHDs: Brominated trihalomethanes: No dose response relationship. Bromodichloromethane exposure during first month of gestation: OR 2.16 (95% CI: 1.05, 4.46).	Subjects were interviewed on water usage in last trimester, not first month, possibly leading to recall bias.

Abbreviations: VSD: Ventricular septal defect.

**Table 5:** Solvents and congenital heart disease: Main findings from the literature.

Author Ref.	Article Type	All CHDs or Any CHD Subtypes	Study Limitations
Watson et al. [48]	Review	Trichloroethylene: All CHDs and All CHDs sub-type: No association	Not a meta-analysis or even a systematic review. Many studies very early.
Hardin et al. [49]	Review	Trichloroethylene and dichloroethylene: All CHDs and any CHD sub-type: no association	As per Watson et al.
Gilboa et al. [50]	Case control study	VSDs: Any solvent OR 1.6 (95% CI: 1.0, 2.6); any chlorinated solvent OR 1.7 (95% CI: 1.0, 2.8). Aortic stenosis: Any solvent: 2.1 (95% CI: 1.1, 4.1). TGA: Stoddart solvent OR 2.0 (95% CI: 1.0, 4.2) RVOTO: Stoddart solvent 1.9 (95% CI: 1.1, 3.3) PVS: Stoddart solvent 2.1 (95% CI: 1.1, 3.8).	Potential exposure misclassification as using solvent classes rather than individual chemicals. Maternal questionnaire-based study so potential recall bias.

Abbreviations: RVOTO: Right ventricular outflow tract obstruction; VSD: Ventricular septal defect; TGA: Transposition of the great arteries; PVS: Pulmonary valve stenosis.

**Table 6:** (**a**) Metals in drinking water or the environment and congenital heart disease: Main findings from the literature. (**b**) Hair and/or placental metals and congenital heart disease: Main findings from the literature.

**(a)**			
**Author Ref.**	**Article Type**	**All CHDs or Any CHD Subtypes**	**Study Limitations**
Zierler et al. [51]	Case control study	All CHDs: No association with any metal. COA: Arsenic OR 3.4 (95% CI: 1.3, 8.9).	Exposure assessed from public water monitor and maternal telephone interview, with possible recall bias. Only limited range of metals tested.
Rudnai et al. [52]	Population based study	Arsenic: All CHDs: OR 1.41 (95% CI: 1.28, 1.56); DBP: OR 1.81 (95% CI: 1.54, 2.11) ASD: OR 1.79 (95% CI: 1.59, 2.01).	Only arsenic tested; CHD incidence compared to other birth defects, so no non-affected control group.
Sanders et al. [54]	Case control study	CTDs: manganese: OR 1.6 (95% CI: 1.1, 2.5). Arsenic, cadmium, lead: No association.	Individual water consumption use not available, so ecological method of assessing exposure used, risking misclassification. Not all potential confounders assessed.
Silver et al. [62]	Case control study	Paternal occupational metal exposure: VSDs: OR 2.70 (95% CI: 1.09, 6.67)	Possible bias towards familial CHDs. Not all potential confounders included.
**(b)**			
**Author Ref.**	**Article Type**	**All CHDs or Any CHD Subtypes**	**Study Limitations**
Jin et al. [55]	Case control study	Hair arsenic: All CHDs: OR 5.62 (95% CI: 3.43, 9.62); Intercardiac defects: OR 6.30 (95% CI: 3.63, 10.92); Extracardiac defects: OR 5.01 (95% CI: 2.42, 10.72) Hair cadmium: Any CHD: OR OR1.96; 95% CI: 1.24, 3.09); Intracardiac defects: OR 1.73 (95% CI: 1.05, 2.86); Extracardiac defects: OR 2.80 (95% CI: 1.40, 5.63)	Hair metal concentrations are only indicative of fetal exposure and are not representative of older exposures, resulting in metals stored in tissue. Maternal interviews can miss information. Concern over hair shampoo altering hair metal levels.
Liu et al. [56]	Case control study	Hair lead: All CHDs: OR 3.07 (95% CI: 2.00, 4.72); All CHDs and other defects: OR 3.55 (95% CI: 1.75, 7.11) Any septal defect: OR 2.06 (95% CI: 1.22, 3.50), CTD: OR 3.66 (95% CI: 2.14, 6.27), RVOTO: OR 3.15 (95% CI: 1.54, 6.43), APVR: OR 3.10 (95% CI: 1.41, 6.86), other heart defects: OR 2.39 (95% CI: 1.16, 4.94)	As per Jin et al.
Hu et al. [57]	Hospital-based case control study	Hair copper: All CHDs: OR 5.70 (95% CI: 2.58, 12.61); CTDs: OR 6.32 (95% CI: 2.11, 18.92)	As per Jin et al., plus small sample size and not representative of the wider population.
Liu et al. [58]	Hospital-based case control study	Hair aluminum: All CHDs: OR 2.32 (95% CI: 1.72, 3.13); All septal defects: OR 2.17 (95% CI: 1.15, 4.10); CTDs: OR, 5.42 (95% CI: 2.43, 2.10); RVOTO: OR 2.43 (95% CI: 1.08, 5.44). LVOTO: No association.	As per Hu et al.
Zhang et al. [59]	Case control study	Hair barium: All CHDs: OR 1.23 (95% CI: 1.146, 1.321) Placental barium: All CHDs: OR 1.392 (95% CI: 1.074, 1.659)	As per Hu et al.

Abbreviations: COA: Coarctation of the aorta; DBP: Ductus Botalli persistens; ASD: Atrial septal defect; VSD: Ventricular septal defect; CTDs: Conotruncal defects; RVOTO: Right ventricular outflow track obstruction; LVOTO: Left ventricular outflow tract obstruction; APVR: Anomalous pulmonary venous return.

**Table 7:** Landfill sites and incinerators and congenital heart disease: Main findings from the literature.

Author Ref.	Article Type	All CHDs or Any CHD Subtypes	Study Limitations
Langlois et al. [65]	Case control study	All CTDs: No association with hazardous waste sites. Truncus arteriosus: Any waste site: OR 2.80 (95% CI: 1.19, 6.54); hazardous waste sites: OR 4.99 (95% CI: 1.26, 14.51)	Used proximity to waste sites at birth date as surrogate for earlier exposure; did not consider CHDs in non-live births or potential for change of residence during pregnancy.
Dummer et al. [66]	Retrospective cohort study	Fatal CHDs: Incinerators: OR: 1.12 (95% CI: 1.03, 1.22)	Used proximity to waste sites at birth date as surrogate for earlier exposure; did not consider CHDs in live births.
Shaw et al. [67]	Population study	All CHDs: Various sources of known contamination: OR 1.1 (95% CI: 1.1, 2.0)	As per Langlois et al.
Croen et al. [68]	Population based case control study	All CHDs: No association with proximity to hazardous waste sites.	As per Langlois et al.
Kuehl et al. [69]	Case control study	TGA: OR 13.4 (95% CI: 4.7, 37.8): Proximity to hazardous waste sites and release of chemicals into the air.	As per Langlois et al.
Dolk et al. [70]	Population-based register study	Residence within 3 km of hazardous industrial waste site: SDs: OR 1.49 (95% CI: 1.09, 2.04); Arterial or venous anomalies: OR 1.81 (95% CI: 1.02, 3.20) No association for malformations of cardiac chambers, valves or other heart defects.	As per Langlois et al. Ignored other exposures.

Abbreviations: CTDs: Conotruncal defects; TGA: Transposition of the great arteries; SDs: Septal defects.

**Table 8:** Persistent organic pollutants and other contaminant and congenital heart disease: Main findings from the literature.

Author Ref.	Article Type	All CHDs or Any CHD Subtypes	Study Limitations
Wang et al. [71]	Case control study	All CHDs: Phthalates: OR 1.6 (95% CI: 1.0, 2.6); Alkylphenolic compounds OR 1.8 (95% CI: 1.1, 3.0)	Exposure assessed by maternal questionnaire, so potential recall bias. Did not assess strength or duration of exposure. Only recent exposure considered. Small size.
Wang et al. [72]	Hospital based case control study	Maternal occupational phthalate exposure: VSD OR 3.7 (95% CI: 1.7, 8.0); PDA OR 3.8 (95% CI: 1.6, 8.9), ASD OR 3.5 (95% CI: 1.4, 8.7); PVS OR 4.2 (95% CI: 1.1, 16.0). Maternal alkylphenolic compound exposure: VSD OR 2.2 (95% CI: 1.3, 3.6); PDA OR 2.0 (95% CI: 1.1, 3.5); PVS OR 3.8 (95% CI: 1.5, 9.4). Maternal heavy metal exposure: VSD OR 7.3 (95% CI: 2.0, 27.6); ASD OR 6.5 (95% CI: 1.1, 36.7). Paternal occupational phthalate exposure: VSD OR 1.6 (95% CI: 1.0, 2.4); PVS OR 2.4 (95% CI: 1.1, 5.2); Paternal alkylphenolic compound exposure: VSD OR 1.5 (95% CI: 1.0, 2.2).	Hospital study not representative of general population, although selection bias minimized. The reference for occupational exposure was developed for Western Europe, which may not be applicable in China. Participants may have recall bias. Non-occupational exposure not assessed.
Liu et al. [73]	Hospital based case control study	All CHDs: Maternal exposure to any housing renovations: OR 1.89 (95% CI: 1.29, 2.77); moved into a new house within one month after decoration at either 3 months before pregnancy (OR 2.38, 95% CI: 1.03, 5.48) or during first trimester (OR 4.00, 95% CI: 1.62, 9.86).	Hospital study not representative of general population. Participants may have recall bias. Not possible to investigate individual CHDs.
Snijder et al. [74]	Case control study	No association of maternal occupational exposure to chemicals with risk of CHDs. Paternal occupational exposure to: Phthalates: All CHDs: OR 2.08 (95% CI: 1.27, 3.40); VSDs: OR 2.84 (95% CI: 1.37, 5.92). PCBs: ASD OR 4.22 (95% CI: 1.23, 14.42); Alkylphenolic compounds: COA OR 3.85 (95% CI: 1.17, 12.67).	Non-occupational exposure not considered. Participants may have recall bias.
Wijnands et al. [75]	Case control study	No association of maternal exposure to chemicals with risk of CHDs. Paternal phthalate exposure: VSD OR 1.93 (95% CI: 1.05, 3.54). No association with paternal exposure to other chemicals.	Did not consider all potentially confounding factors. Possible recall and other bias.

Abbreviations: VSD: Ventricular septal defect; ASD: Atrial septal defect; PDA: Patent ductus arteriosus; PVS: Pulmonary valve stenosis.
